# Mediating Effects of Self-Efficacy and Illness Perceptions on Mental Health in Men with Localized Prostate Cancer: A Secondary Analysis of the Prostate Cancer Patient Empowerment Program (PC-PEP) Randomized Controlled Trial

**DOI:** 10.3390/cancers16132352

**Published:** 2024-06-27

**Authors:** Cody MacDonald, Gabriela Ilie, George Kephart, Ricardo Rendon, Ross Mason, Greg Bailly, David Bell, Nikhilesh Patil, David Bowes, Derek Wilke, Andrea Kokorovic, Robert D. H. Rutledge

**Affiliations:** 1 Department of Urology, Faculty of Medicine, Dalhousie University, Halifax, NS B3H 2Y9, Canada; 2 Department of Community Health and Epidemiology, Faculty of Medicine, Dalhousie University, Halifax, NS B3H 1V7, Canada; 3 Department of Radiation Oncology, Faculty of Medicine, Dalhousie University, Halifax, NS B3H 1V7, Canada

**Keywords:** prostate cancer, mental health, empowerment, patient activation, patient education, behavioural intervention, survivorship, self-efficacy, illness perception, quality of life

## Abstract

**Simple Summary:**

This study investigates how the Prostate Cancer Patient Empowerment Program (PC-PEP) helps men with prostate cancer by promoting patient empowerment and activation. This program encourages patients to engage actively in their care, fostering healthy living habits daily, over six months. Involving 128 patients, this research found that those participating in the PC-PEP experienced significant improvements in self-efficacy and emotional responses, leading to reduced psychological distress compared to those receiving standard care. These findings underscore the importance of integrating the PC-PEP into clinical practice to enhance mental health and patient care strategies.

**Abstract:**

Understanding how interventions reduce psychological distress in patients with prostate cancer is crucial for improving patient care. This study examined the roles of self-efficacy, illness perceptions, and heart rhythm coherence in mediating the effects of the Prostate Cancer Patient Empowerment Program (PC-PEP) on psychological distress compared to standard care. In a randomized controlled trial, 128 patients were assigned to either the PC-PEP intervention or standard care. The PC-PEP, a six-month program emphasizing daily healthy living habits, included relaxation and stress management, diet, exercise, pelvic floor muscle exercises, and strategies to improve relationships and intimacy, with daily activities supported by online resources and live sessions. Participants in the intervention group showed significant improvements in self-efficacy and specific illness perceptions, such as personal control and emotional response, compared to the control group. These factors mediated the relationship between the intervention and its psychological benefits, with self-efficacy accounting for 52% of the reduction in psychological distress. No significant differences in heart rhythm coherence were observed. This study highlights the critical role of self-efficacy and illness perceptions in enhancing psychological health in prostate cancer patients through the PC-PEP. The results underscore this program’s effectiveness and the key mechanisms through which it operates. Given the high rates of distress among men undergoing prostate cancer treatments, these findings emphasize the importance of integrating the PC-PEP into clinical practice. The implementation of the PC-PEP in clinical settings can provide a structured approach to reducing psychological distress and improving overall patient well-being.

## 1. Introduction

Prostate cancer is the most common cancer in men in 112 countries, accounting for 15% of all male cancers [1,2]. According to a recent Lancet Commission, the number of new prostate cancer cases annually is projected to rise from 1.4 million in 2020 to 2.9 million by 2040, driven by demographic changes and increased life expectancy [3]. Despite the high survival rates of prostate cancer compared to other malignancies, the quality of life of survivors remains a critical concern due to the substantial prevalence of depression, suicide, and psychological distress, which can persist long after diagnosis [4,5,6,7,8]. This underscores the necessity for effective interventions that alleviate the psychological impacts of prostate cancer from diagnosis through long-term survivorship.

A growing body of research indicates that lifestyle self-management interventions tailored for prostate cancer can enhance mental health and quality of life [9,10,11]. The Prostate Cancer Patient Empowerment Program (PC-PEP) is one such intervention, offering a multifaceted approach that prioritizes various aspects of well-being, including physical activity, healthy nutrition, sleep hygiene, relaxation/stress-reduction techniques, pelvic floor muscle training, human connection, and peer support. The program content is disseminated through daily emails and videos, as well as live monthly videoconferences over six months, providing consistent encouragement and support. The efficacy of the PC-PEP in reducing psychological distress, depression, and anxiety compared to standard care has been previously reported [12]. However, the specific mechanisms or pathways underlying this relationship remain to be fully elucidated [13].

Self-management encompasses the day-to-day activities individuals undertake to manage their conditions, recognizing people as experts in their own care [14,15]. To gain the skills and knowledge to self-manage most effectively, patients often need the support of others [16]. The PC-PEP equips prostate cancer patients with knowledge, skills, and support, all crucial elements for productive self-management. Moreover, social cognitive theory and the common-sense model of self-regulation (CSM) posit that an enhancement in self-efficacy and illness perceptions, respectively, is essential for achieving effective self-management that leads to desired outcomes, such as improved mental health [17,18].

Self-efficacy is defined as the belief in one’s capacity to execute behaviors necessary to produce specific outcomes [19]. Greater self-efficacy has been associated with better management of cancer-related side effects and challenges [20,21]. An inverse relationship between self-efficacy and psychological distress in cancer patients has been established, suggesting that enhancing self-efficacy could be a key strategy in alleviating psychological distress [11,20]. Self-efficacy can be increased through mastery experiences, social modeling, and social persuasion [22], all of which are targeted in the PC-PEP. For example, participants are challenged to set goals and engage in progressively more challenging exercises throughout the program (mastery experiences). They are encouraged to connect with other participants and mentors weekly (social modeling), and they receive daily encouragement and motivational messages (social persuasion) to reinforce and maintain their newly acquired healthy habits or to adopt additional healthy behaviors. The PC-PEP also incorporates various technologies, including daily emails and videos, text message reminders, website and online resources, and a biofeedback device used in conjunction with a smartphone app. DiClemente et al. [23] recommend that health-promoting programs better utilize today’s widespread technologies to have greater success at influencing behavioral changes and delivering more tailored interventions that better meet patients’ needs. While self-management programs in general have been shown to improve self-efficacy, those incorporating new technologies, such as the PC-PEP, can break down barriers to access and help sustain behavioral changes, leading to greater success in improving self-efficacy [24].

The CSM highlights the importance of individual illness perceptions in self-management [25,26]. These perceptions include views on the consequences, timeline, personal and treatment control, identity, cause, and emotional impact of the illness. Negative illness perceptions have been significantly associated with psychological distress and a reduced quality of life in cancer patients [27,28,29,30]. Therefore, modifying illness perceptions may be a viable approach to improving health outcomes. The components of the PC-PEP provide an avenue for patients to gain a greater sense of personal control over the impacts of their prostate cancer diagnosis and acquire knowledge that could influence how they perceive various aspects of the disease. While there are many commonalities between the CSM and self-efficacy frameworks, the integration and use of both frameworks have been supported to better understand self-management and improve health outcomes [31].

An additional component of the PC-PEP intervention is heart rate variability (HRV) training using a biofeedback device from HeartMath Inc. (Boulder Creek, California, USA), aimed at improving heart rhythm coherence—a physiological state of heart rate pattern synchronization [32,33]. While this training has been associated with reduced stress and better emotional regulation [32,34], its impact on heart rhythm coherence and mental health outcomes in a comprehensive lifestyle intervention like the PC-PEP remains to be thoroughly investigated, as indicated by mixed results in smaller-scale studies [35,36].

Considering these factors, we hypothesized that self-efficacy, illness perceptions, and, to a lesser extent, heart rhythm coherence, serve as predictors of psychological distress and partially account for the observed effectiveness of the Prostate Cancer Patient Empowerment Program (PC-PEP) in mitigating it. Investigating these variables under varying conditions, such as different treatment modalities for prostate cancer, could provide deeper insights into the mechanisms of action [37,38]. Therefore, this secondary analysis of the PC-PEP trial aims to assess whether the intervention (A) enhances self-efficacy, illness perceptions, and heart rate coherence; (B) reduces psychological distress through these factors (mediation); and (C) varies in its effectiveness based on the patient’s treatment modality (moderated mediation). 

## 2. Materials and Methods

This secondary analysis utilizes data from the Prostate Cancer Patient Empowerment Program (PC-PEP) trial, a single-center, prospective, delayed crossover, randomized controlled trial conducted between December 2019 and January 2021 in Halifax, Nova Scotia. The trial assessed 171 men diagnosed with prostate cancer (PC) and scheduled for curative treatment, comparing the effects of the PC-PEP with standard care. Participants provided informed consent, and the study received ethical approval from the Nova Scotia Health Authority [1024822, https://ClinicalTrials.gov (accessed on 26 June 2024) NCT03660085], adhering to the Declaration of Helsinki. The research was conducted in accordance with the CONSORT reporting guidelines, with the detailed study protocol documented elsewhere [12].

Participants were recruited from the Departments of Urology and Radiation Oncology at the Queen Elizabeth II Health Sciences Centre in Halifax, Nova Scotia, Canada, from December 2019 to January 2021. Advertisements were also shared with PC support groups, and self-referrals were accepted from interested participants throughout the Maritime provinces of Canada. Eligibility criteria included the following: a biopsy-proven PC diagnosis; age 18 or older; scheduled to receive curative treatment (either radical prostatectomy or primary or salvage radiation therapy) within 6 months of enrollment; approval from the study physician for the safety and ability to participate in low-to-moderate physical activity; an email address, computer/smartphone/tablet, and daily internet access to receive the intervention; able to read and understand English; a controlled systolic blood pressure of <160 and a diastolic pressure of <90; and willing to travel to the study centre for 3 study visits (at baseline, 6 months, and 12 months). 

**Figure 1:**
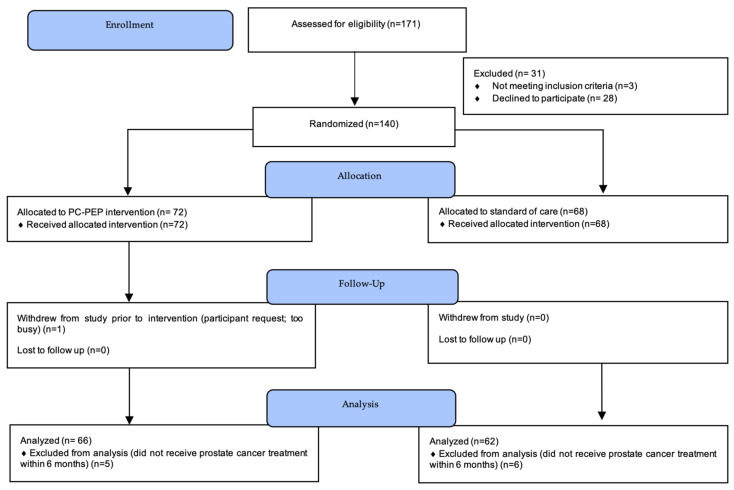
CONSORT 2010 flow diagram. CONSORT = Consolidated Standards of Reporting Trials; PC-PEP = Prostate Cancer Patient Empowerment Program.

Figure 1 illustrates the progression of participants through the study, adhering to the CONSORT 2010 guidelines. Initially, 171 potential participants were screened, 3 of which were ineligible and 28 chose not to participate, leading to a total of 140 participants being randomly allocated to either the intervention or the control group. Post-allocation, one participant withdrew before starting the intervention. An additional 11 participants were excluded from the analysis, as they did not undergo curative treatment within 6 months of enrollment, thereby not meeting the post-randomization criteria for inclusion in the study.

Participants meeting the eligibility criteria who provided informed consent were asked to complete an online baseline study survey, managed using Research Electronic Data Capture (REDCap), hosted at Nova Scotia Health [39]. After completion of the baseline survey, participants attended an in-person baseline study visit to collect all remaining physical measures, including the HRV outcomes. Data were collected by a trained research coordinator and certified exercise physiologist. Participants were then randomly allocated to the intervention or waitlist control group.

Using a fixed block design, randomization was stratified by the presence of psychological distress (Kessler Psychological Distress Scale [K10] ≥ 20 or <20) at the baseline; the curative treatment type (radical prostatectomy, radiation therapy, or salvage radiation post-surgery); and the presence or absence of hormone therapy in the patient’s treatment plan. This randomization sequence was kept concealed from the research staff responsible for data collection and participant management. Participants were then scheduled for follow-up study visits at the 6-month mark to assess progress and collect further data.

### 2.1. Intervention

The intervention and protocol of the PC-PEP randomized trial are described in detail elsewhere [12]. Briefly, participants assigned to the intervention were sent an automated email daily for 6 months, containing a 3–5 min video message from the principal investigator (GI) and study physician (RR) that promoted healthy lifestyle behaviours, provided daily encouragement, and prescribed the intervention activities. 

Key components of the intervention included the following (A) *relaxation/stress reduction*—participants were provided a heart rate variability biofeedback device (the Inner Balance or emWave2 by HeartMath Inc.) and asked to use it daily by either following a 10 min guided mindfulness meditation video or the Quick Coherence^®^ technique (HeartMath Inc., n.d.) that encourages slow, deep breathing matched to a breath pacer, along with a sincere attempt at experiencing regenerative feelings such as appreciation or care; (B) *nutrition*—dietary behaviour changes were encouraged, such as increasing fruit and vegetable intake and reducing red meat; (C) *physical activity*—participants are encouraged to exercise daily to reach a minimum of 150–300 min per week of aerobic activity and at minimum 2 days of resistance strength training; (D) *pelvic floor muscle training*—participants were educated by a pelvic floor nurse specialist and asked to follow guided videos for 10 min, 3 times per day that were progressively more challenging each week; (E) *relationships and intimacy*—recommendations on addressing intimacy and sexuality issues, as well as tips to improve connections and relationships, were made. An optional one-hour social support videoconference was also hosted monthly, and participants could opt in to connect with two co-participants and were encouraged to call each other weekly as a form of peer-to-peer patient support. Participants also received weekly compliance surveys to report minutes of each activity and to encourage adherence.

### 2.2. Non-Specific Psychological Distress

Non-specific psychological distress was assessed using the Kessler Psychological Distress Scale (K10) [40]. The K10 comprises 10 items, each of which is rated from 1 to 5, on a 5-point Likert Scale, to assess symptoms of depression and anxiety in the last month. The summary score can range from 10 to 50, with higher scores indicating worse psychological distress [41]. Cut-off points have been established, with a total score of <20 indicating no significant psychological distress, 20–24 indicating mild distress, 25–29 indicating moderate distress, and 30–50 indicating severe levels of distress [41]. The use of the K10 in cancer patients has been found to have good internal consistency (Cronbach’s alpha = 0.914) [42].

### 2.3. Mediator Variables

*Self-efficacy* was evaluated using the Self-Efficacy for Managing Chronic Diseases 6-item scale (SEMCD-6) [43]. This scale employs a 10-point Likert format, where response options for each item range from “not at all confident” (1) to “totally confident” (10). The SEMCD-6 scale has demonstrated high internal consistency in other studies, with Cronbach’s alpha values ranging from 0.88 to 0.91, and it is sensitive to detecting changes over time [44]. The total score is calculated by averaging the scores of all items, with higher averages indicating greater self-efficacy. The Cronbach’s alpha coefficient for our sample was 0.926, suggesting good internal consistency. 

*Illness perceptions* were assessed using the Brief Illness Perception Questionnaire (B-IPQ) [25]. The B-IPQ includes 8 items, each of which captures a unique, individual dimension of illness perception, on a linear 0-to-10-point response scale. Higher scores represent a more negative perception of an illness for 5 of the items [(1) consequences, (2) timeline, (5) identity, (6) coherence, and (8) concern] and a more positive perception on 3 items [(3) personal control, (4) treatment control, and (7) emotional representation]. While the domains of illness perceptions were analysed using their single item, a continuous composite score was also obtained by reversing the 3 latter items and adding all scores together. Higher composite scores on the B-IPQ indicate that a person overall feels more threatened by the illness [25]. However, the composite score of the B-IPQ may not be valid for certain illnesses, depending on how the unique items are related, and thus must be reported with caution [45]. The B-IPQ has been found to have acceptable predictive and discriminant validity, as well as good test-retest reliability, across multiple language versions and populations [45]. It has been shown that the B-IPQ has good concurrent validity with the longer IPQ-Revised, with respect to quality of life, and it is sensitive to change [44]. The tool has also shown to be reliable and valid in measuring illness perceptions in various medical conditions [46,47]. Pearson’s correlations for test-retest reliability were generally acceptable (with a range of 0.5–0.7) [25]. For our sample, the Cronbach’s alpha coefficient was 0.488, suggesting poor internal consistency.

Data for *heart rhythm coherence* were captured using the Inner Balance (wired model) biofeedback device produced by HeartMath Inc. This instrument uses HRV to derive readings of heart coherence by analyzing the highest peak in the 0.04 Hz to 0.26 Hz range of the HRV power spectrum, followed by a calculation of the integral in a 0.030 Hz-wide window that is centered on the maximum peak, and, lastly, calculating the power of the full spectrum. Participants underwent a 10 min data collection session that captured the average coherence score for the session. A higher coherence score represents a more coherent heart rhythm and is associated with lower stress and a more positive emotional state [32,48].

### 2.4. Prognostic Covariates

Drawing from the existing literature on variables associated with the study outcomes [49,50,51,52], the subsequent variables were selected a priori as predictive covariates: age (continuous); the prescribed medication for depression, anxiety, or both (coded as 1 for yes and coded as 0 for no); relationship status (coded as 1 for in a relationship and coded as 0 for single); the number of days between the start of treatment and the date of randomization (continuous); the type of curative treatment received (coded as 1 for radical prostatectomy and coded as 2 for primary or salvage radiation therapy); and the Charlson Comorbidity index score. These chosen variables were incorporated as covariates due to their demonstrated prognostic relevance for mental health outcomes in the prostate cancer population and alignment with prior research findings.

### 2.5. Statistical Analysis

Statistical analyses were conducted using SPSS version 28.0 [53]. We report baseline demographic characteristics using descriptive statistics, presenting continuous variables as means and standard deviations and categorical variables as frequencies and percentages. To compare baseline characteristics between groups, we employed independent samples *t*-tests for continuous variables and chi-square (χ^2^) or Fisher Exact tests (for small cell counts) for categorical variables. A *p*-value of less than 0.05 (two-sided) was considered statistically significant. To evaluate the impact of the intervention on non-specific psychological distress, self-efficacy, illness perceptions (including individual domains and composite score), and heart rhythm coherence at 6 months, we performed separate one-level linear mixed modeling analyses with an unstructured covariance structure, controlling for baseline scores for each outcome alongside prognostic covariates. Statistically significant associations identified through this step were then subjected to mediation analyses using the PROCESS macro, version 4.2, for SPSS, model 4 [54]. The PROCESS macro uses a traditional mediation method with ordinary least squares regression [54]. This method has been found to have equivalent effect estimates as newer, causal methods, in relatively simple mediation analyses with continuous mediators and outcomes [55], and traditional approaches remain the most utilized and reported method of mediation analyses in randomized controlled trials [56]. The PROCESS macro calculates regression coefficients for four pathways (a, b, c′, and c) within the mediation framework. Pathway ‘a’ examines the relationship between the independent variable (group assignment) and the potential mediator at 6 months (while controlling for baseline mediator scores and prognostic covariates), whereas pathway ‘b’ assesses the relationship between the potential mediator at 6 months and the outcome (psychological distress) at 6 months, independent of the group, while controlling for the baseline measurement for the mediator and the outcome and baseline prognostic covariates. Pathways c’ and c represent the direct and total effect of the association between the PC-PEP and psychological distress at 6 months, respectively. The indirect effect (mediation effect) is determined by multiplying coefficients from pathways’ ‘a’ and ‘b’, with its statistical significance assessed using bootstrapped confidence intervals. We chose 95% confidence intervals with 10,000 bootstrap resamples. An indirect effect’s confidence interval that does not include zero indicates significant mediation. The proportion of the effect that was mediated was calculated by dividing the regression coefficient of the indirect effect by that of the total effect to obtain a percentage.

Moderated mediation analyses, using the PROCESS macro, model 7, investigated whether treatment modality (radical prostatectomy or radiation therapy) modified the mediation effects. Baseline scores for both the outcome and mediator, along with other prognostic covariates, were included as covariates to adjust for pre-intervention effects. All data from self-reported questionnaires were complete, with no missing data. However, due to COVID-19 restrictions that limited some in-person study visits, approximately 11% of the heart rhythm coherence data was missing at the baseline and 25% at 6 months. We conducted complete case analyses for these measures, which led to the exclusion of 37 cases from these specific analyses.

## 3. Results

Participants in the sample were predominantly white, retired, and married or in a relationship. At the baseline, the average age of the participants was 66 years, ranging from 50 to 82 years, and the group was predominantly overweight, with a mean Body Mass Index (BMI) of 29.9 (SD = 6.36). Approximately half of the sample received radical prostatectomy during the study while the other half received radiation therapy or salvage radiation following biochemical recurrence after a previous radical prostatectomy. There were no statistically significant differences in the baseline characteristics observed between the PC-PEP and waitlist control groups (Table 1).

### 3.1. Mediation Analyses

#### 3.1.1. Predictor to Outcome: Effects of Group (Intervention vs. Usual Standard of Care) on Psychological Distress

A linear modeling analysis revealed a statistically significant difference between the groups (control vs. the PC-PEP) in non-specific psychological distress at 6 months (*p* = 0.013), with the mean difference showing higher psychological distress for the control group at 6 months compared to the PC-PEP group (Table 2), while controlling for the baseline psychological distress and prognostic covariates. 

#### 3.1.2. Predictor to Mediators: Effects of Group (PC-PEP vs. Usual Standard of Care) on Potential Mediating Variables

Linear modeling analyses revealed significant differences in self-efficacy levels at the 6-month follow-up, controlling for baseline scores and prognostic covariates. The control group exhibited significantly lower self-efficacy compared to the PC-PEP intervention group (*p* = 0.023; see Table 2). The Cronbach’s alpha coefficient was 0.926, indicating high internal consistency.

For the illness perception domains, the analysis revealed that participants in the control group perceived significantly less personal control over their prostate cancer diagnosis (*p* = 0.032) and exhibited more negative emotional representations (*p* = 0.026) compared to those in the intervention group, as detailed in Table 2. These results indicate that the intervention positively impacted participants’ perceived control and emotional responses to their diagnosis. No other domains within the illness perception measure showed significant differences between the groups at the 6-month mark, and the composite sum score of illness perceptions did not reveal a significant difference between the groups (*p* = 0.085).

There were no significant differences in heart rate variability (HRV) coherence or HRV achievement outcomes between the intervention and control groups at 6 months.

#### 3.1.3. Tests of the Mediation Pathways

**Figure 2:**
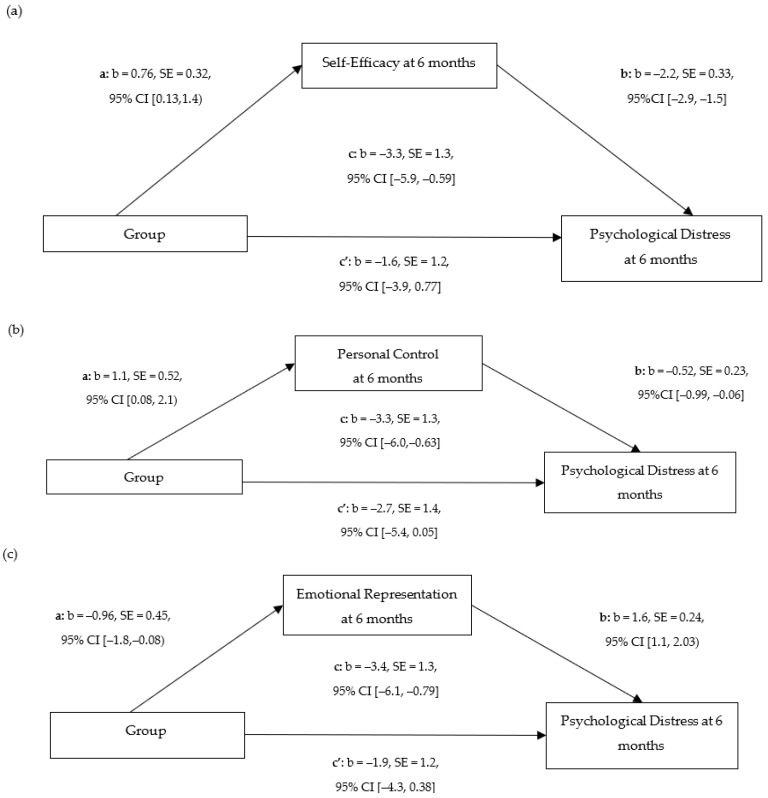
Mediation model including group as the predictor; psychological distress as the outcome; and (**a**) self-efficacy, (**b**) personal control, and (**c**) emotional representation as mediators. Results were obtained using the PROCESS macro for SPSS, model 4. c = total effect; c′ = direct effect; b = standardized regression coefficient.

Figure 2 presents the estimated path coefficients derived from bootstrapped simple mediation analyses, conducted using ordinary least squares path analyses through the PROCESS macro (model 4). These analyses investigated the mediating effects of three variables found to be statistically associated with group in the previous linear modelling analyses, on the relationship between group assignment (intervention vs. control) and the outcomes of psychological distress and the need for clinical treatment at the 6-month follow-up. Specifically, the analyses explored the mediating roles of (Figure 2a) self-efficacy; (Figure 2b) personal control, as measured by the Brief Illness Perception Questionnaire (B-IPQ) personal control; and (Figure 2c) emotional representation, as assessed by the B-IPQ emotional representation, all evaluated at 6 months. This structure allowed for a nuanced examination of how each potential mediator influences the intervention’s effects on the outcomes.

The mediation analyses revealed that all paths from the group to the mediators (path a) and from the mediators to the outcome (path b) were statistically significant, as illustrated in Figure 2. The indirect effects of self-efficacy, B-IPQ personal control, and B-IPQ emotional representation on the relationship between group allocation (PC-PEP vs. control) and outcomes at 6 months were statistically significant, which was validated by bootstrap confidence intervals. This evidence supports the mediation hypothesis for these variables, as detailed in Table 3.

Moreover, the direct effect of the Brief Illness Perception Questionnaire (B-IPQ) personal control on the outcome was statistically significant (*p* = 0.046). However, the direct effects for self-efficacy (*p* = 0.19) and B-IPQ emotional representation (*p* = 0.099) were not statistically significant. The increase in self-efficacy from the baseline to 6 months accounted for over half (52%) of the intervention’s effect. In separate mediation models, changes in B-IPQ personal control and emotional representation contributed approximately 18% and 44% to the intervention’s overall effect, respectively, as detailed in Table 3.

### 3.2. Moderated Mediation Analyses

The moderated mediation analyses investigating the effect of prostate cancer treatment modality (surgery vs. radiation) on the mediation process revealed no significant effects for any of the mediators. This conclusion is supported by the bootstrapped confidence intervals presented in Table 4, indicating that the treatment modality did not influence the mediation relationships.

## 4. Discussion

This secondary analysis of the Prostate Cancer Patient Empowerment Program (PC-PEP) randomized controlled trial aimed to investigate how changes in self-efficacy, illness perceptions, and heart rhythm coherence mediate the relationship between group assignment (PC-PEP intervention or usual standard of care) and reductions in psychological distress and the need for clinical treatment. Consistent with our hypotheses and supporting the literature [11,57,58,59], the PC-PEP intervention significantly improved participants’ self-efficacy, perceptions of personal control, and emotional responses related to their illness. These improvements align with the concept that self-management interventions can enhance outcomes by empowering patients to manage their health and treatment side effects more effectively.

The sample predominantly consisted of white, retired individuals in a relationship, with an average age of 66 years and a tendency towards being overweight. This demographic profile, along with the balanced distribution of treatment modalities (radical prostatectomy and radiation therapy), provides a specific context in which the intervention’s effects were observed. No significant baseline differences between the PC-PEP and control groups were noted, indicating that any post-intervention differences can more confidently be attributed to the intervention itself.

Our findings reveal that the PC-PEP notably improved participants’ sense of personal control and reduced negative emotional representations associated with their prostate cancer diagnosis. However, the PC-PEP did not significantly impact the overall illness perceptions’ composite score. The internal consistency for this score was very low, and as indicated by Broadbent et al. [45], the composite score may not be valid in the context of all illnesses, depending on how the domains are related in their specific contexts. Therefore, the use of this score is likely less useful for clinical applications in prostate cancer, as illness perception domains are unique, and information is lost when domains are analyzed together, leading to low consistency among items.

The results suggest that the PC-PEP’s focus on lifestyle modifications and promoting healthy behavioral changes successfully empowered participants, enhancing their self-efficacy and perception of personal control. However, the program’s lesser emphasis on the informational aspects of prostate cancer and its treatments may explain why other illness perception domains were not significantly affected. This indicates that improving one’s knowledge or understanding of the disease and treatment mechanisms might require additional or different educational components within the intervention framework. Evidence suggests that enhancing patient understanding and empowerment through materials such as YouTube™ videos and other social media channels may be beneficial [60]. However, a recent study found that while 52% of YouTube™ videos on prostate cancer were created by physicians, the overall content was often neither exhaustive nor reliable, highlighting a general underestimation of the mental health needs of prostate cancer patients. This underscores the importance of producing and presenting educational materials by qualified professionals—such as in the case of the PC-PEP, a prostate cancer radiation oncologist, and a scientist with over 25 years of experience—to ensure accuracy and reliability. Consequently, a multidisciplinary agreement to establish quality standards and improve communication about mental health care is essential.

Contrary to the positive outcomes observed in self-efficacy and certain illness perceptions, heart rhythm coherence outcomes did not significantly change following the intervention. This finding is consistent with the PC-PEP pilot study [35] and contrasts with other research indicating potential benefits of HRV biofeedback training [36]. The multifaceted nature of the PC-PEP and a lower adherence to the HRV training component suggest that participants might have derived more benefits from other aspects of the intervention, such as physical activity and dietary changes. This raises questions about the utility of the HRV device within this intervention context, suggesting that omitting it could streamline program integration and enhance its cost-effectiveness.

The mediation analyses underscored the significant roles of self-efficacy, B-IPQ personal control, and B-IPQ emotional representation in mediating the intervention’s effects on psychological distress, with variations in their contributions. Notably, the moderated mediation analyses did not reveal significant effects of prostate cancer treatment modality on these mediation processes, suggesting that the intervention’s benefits transcend these treatment distinctions.

This study provides valuable insights into the mechanisms by which the PC-PEP intervention reduces psychological distress among prostate cancer patients, underscoring the significance of bolstering self-efficacy and altering illness perceptions. Conducted within a randomized controlled trial framework, this research provides robust evidence to substantiate these effects. It is noteworthy, however, that the completeness of the data sets varied across measures. While survey-based measures benefitted from complete data sets, approximately 29% of the participants were excluded from analyses concerning heart rhythm coherence data due to missing information. This exclusion suggests that, although the findings related to self-efficacy and illness perceptions are based on complete data, the estimates related to heart rhythm coherence may be biased. Therefore, while the study presents strong evidence for the intervention’s efficacy in certain areas, caution is advised in interpreting results related to heart rhythm coherence.

The analyses presented here are secondary in nature, and the original trial was not designed with the statistical power to assess changes in self-efficacy, illness perceptions, or heart rhythm coherence. This limitation is significant, as it may affect the robustness of findings in these specific areas. As secondary analyses, post hoc power estimates are not recommended [61]. Nonetheless, post hoc exploratory analyses are still important for generating new knowledge and encouraging future prospective studies that primarily consider these variables.

The measure used to assess self-efficacy was designed for various chronic diseases, and thus a disease-specific measure might have better captured the construct of self-efficacy in the context of prostate cancer. Despite this, the measure’s internal consistency was high (α = 0.926). The measure used to assess illness perceptions also poses some limitations. The low internal consistency observed indicates that the items may measure different constructs, reducing the reliability of the overall composite score. The strength of this relationship may have been underestimated, requiring a cautious interpretation of the findings. Although previous research found concurrent validity with the longer IPQ-Revised [24], studies were not conducted on prostate cancer patients, and the single items may not fully capture the complexities of each domain. Thus, the content validity of the measure may be reduced, again reducing the reliability of the results and weakening the observed relationships.

Furthermore, the mediator variables and outcomes were both assessed at the same time points (pre- and post-intervention). This limits our ability to understand the temporal relationships among variables and weakens our ability to establish causality from the mediator to the outcome. Despite this limitation, our assumptions about the causal sequence are grounded in underlying theoretical frameworks, including the common-sense model of self-regulation (CSM) and social cognitive theory, which suggests that illness perceptions and self-efficacy precede health outcomes like psychological distress [17,18]. These analyses serve as an initial exploration within the context of the PC-PEP intervention, offering insights for future research that should assess mediators at multiple or intermediate time points throughout the intervention.

Both the self-efficacy scale item and the B-IPQ item for the emotional representation domain assess aspects related to emotional distress, albeit from slightly different perspectives. These mediators specifically may have a conceptual overlap with the outcome, psychological distress. While the Kessler Psychological Distress Scale assesses symptoms of depression and anxiety, these concepts remain interconnected, potentially inflating observed mediation effects. Particularly, the role of the B-IPQ emotional representation domain, which focused solely on emotional impact and was captured by a single item, may be overemphasized in these analyses.

The use of Hayes’ method of mediation in these analyses offers advantages over traditional approaches, such as the method by Baron and Kenny [62]. Particularly, PROCESS employs bootstrapping for obtaining confidence intervals to assess statistical significance, which is suitable for the sample sizes in this study [54]. Nonetheless, more advanced methods, such as those that use structural equation modeling, provide a more comprehensive framework to mediation analyses, allowing the explicit modeling of measurement errors and handling missing data more effectively [63]. Despite its limitations, Hayes’ method remains valuable and widely used, especially in exploratory contexts like this study. This study opted for separate single mediator models instead of a multiple mediator model, which may oversimplify the mediation pathways and overlook the interrelationships among mediators. Despite this limitation, this is the first study to explore these mediation pathways in the context of the PC-PEP, and single mediation models allow us to examine the independent role of each mediator, offering insights into their individual contributions to reducing psychological distress.

Moreover, the recruitment strategy, which relied on volunteer participation and convenience sampling at a single center, may introduce potential volunteer biases. These biases, along with the study’s single-center setting, may restrict the generalizability of our results to broader and more diverse populations. To overcome these limitations and enhance the validity and applicability of our findings, future research efforts should focus on multi-center trials [currently underway, https://www.pcpep.org (accessed on 26 June 2024)]. These expanded studies would not only provide a more powerful analysis of the intervention’s effects on self-efficacy, illness perceptions, and heart rhythm coherence but also ensure that the results are representative of a wider patient demographic.

Clinically, identifying patients with low self-efficacy or perception of personal control may enable healthcare providers to tailor recommendations for PC-PEP participation more effectively. Integrating such psychological prehabilitation into a multimodal approach [64] could significantly enhance patient support during their cancer journey, potentially mitigating psychological distress proactively.

In conclusion, the PC-PEP intervention demonstrates a promising strategy for reducing psychological distress in prostate cancer patients by improving self-efficacy, personal control, and emotional representation perceptions. These findings underscore the potential of similar interventions to enhance patient outcomes and support their implementation in clinical practice.

## 5. Conclusions

In conclusion, the PC-PEP intervention demonstrates a promising strategy for reducing psychological distress in prostate cancer patients by improving self-efficacy, personal control, and emotional representation perceptions. These findings underscore the potential of similar interventions to enhance patient outcomes and support their implementation in clinical practice. Given the significant improvements observed in self-efficacy and emotional response, clinicians should consider incorporating self-management and empowerment programs like the PC-PEP as part of standard care for prostate cancer patients. Tailoring interventions to focus on both lifestyle modifications and enhancing patients’ knowledge and understanding of their disease could further improve other aspects of illness perceptions that were not significantly impacted in this study.

For clinical care, it is crucial to identify patients with low self-efficacy or perceptions of personal control early in their treatment journey. Healthcare providers can then recommend participation in empowerment programs like the PC-PEP to these individuals, potentially mitigating psychological distress more effectively. Integrating psychological prehabilitation into a multimodal approach to cancer care can provide comprehensive support, addressing both physical and mental health needs. Additionally, considering the limited impact of the HRV component in this study, future implementations of the PC-PEP might benefit from focusing on more impactful elements such as physical activity and dietary changes, thereby streamlining the program and enhancing cost-effectiveness. Multi-center trials are essential to validate these findings and ensure that they are representative of a broader patient demographic, ultimately leading to more personalized and effective care strategies for prostate cancer patients.

## Figures and Tables

**Table 1:** Baseline sample characteristics of participants in the intervention and control groups in the Prostate Cancer Patient Empowerment Program (PC-PEP) trial.

	PC-PEP (n = 66)	Control (n = 62)	*p*-Value
Age (years)	65 (6.8)	67 (7.2)	0.7
Race			0.1
White	60 (91%)	61 (98%)	
Black	4 (6.1%)	0 (0%)	
Latino	1 (1.5%)	0 (0%)	
Middle Eastern	1 (1.5%)	0 (0%)	
Other	0 (0%)	1 (1.6%)	
Education			0.4
Less than high school	8 (12%)	6 (9.7%)	
High school or college diploma	27 (41%)	19 (31%)	
University degree	31 (47%)	37 (60%)	
Relationship status			0.063
In a relationship	59 (89%)	61 (98%)	
Not in a relationship	7 (11%)	1 (1.6%)	
Employment status			0.7
Working full-time	17 (26%)	19 (31%)	
Working part-time	5 (7.6%)	4 (6.5%)	
Retired	43 (65%)	39 (63%)	
Unemployed	1 (1.5%)	0 (0%)	
Annual household income			0.7
<CAD 30,000	12 (18%)	10 (16%)	
CAD 30,000–CAD 79,999	22 (33%)	16 (26%)	
CAD 80,000–CAD 100,000	12 (18%)	15 (24%)	
>CAD 100,000	20 (30%)	21 (34%)	
Treatment modality			0.065
Radical prostatectomy (RP)	29 (44%)	33 (53%)	
Radiation therapy	27 (41%)	27 (44%)	
Salvage radiation, post-RP	10 (15%)	2 (3.2%)	
Prescribed hormone therapy	27 (40%)	21 (34%)	0.4
Prescribed medication for anxiety and/or depression	12 (18%)	7 (11%)	0.3
Body mass index	30.8 (6.8)	29.0 (5.7)	0.1
Charlson Comorbidity index	0.36 (0.69)	0.39 (0.58)	0.8

Note: Data are presented as means with standard deviation (SD) or n (%).

**Table 2:** Observed means (SDs) and estimated group (control vs. PC-PEP) mean differences at 6 months in primary outcome and potential mediating variables.

	Baseline Observed Mean (SD)		6 Months Observed Mean (SD)		6-Month Mean Adjusted Difference * (95% CI)	*p*
	Control	PC-PEP	Control	PC-PEP		
Psychological distress (K10)	15 (4.1)	15 (5.2)	17 (9.6)	14 (4.9)	3.4 (0.73, 6.007)	0.013
Self-efficacy	7.3 (1.6)	7.6 (1.6)	7.6 (2.2)	8.4 (1.7)	−0.75 (−1.4, −0.103)	0.023
Illness perceptions						
Consequences	3.3 (2.7)	3.1 (2.8)	4.2 (2.8)	3.3 (2.5)	0.78 (−0.106, 1.7)	0.084
Timeline	4.8 (3.3)	4.3 (2.9)	4.3 (3.6)	4.2 (3.8)	−0.15 (−1.4, 1.1)	0.8
Personal control	3.9 (3.1)	4.7 (2.6)	3.8 (3.1)	5.4 (2.9)	−1.2 (−2.2, −0.102)	0.032
Treatment control	8.6 (1.7)	9.0 (1.3)	8.1 (2.6)	8.7 (1.9)	−0.74 (−1.6, 0.09)	0.080
Identity	1.9 (2.1)	1.9 (2.1)	3.5 (2.6)	2.6 (2.4)	0.83 (−0.093, 1.8)	0.078
Concern	6.1 (3.1)	6.4 (3.1)	4.2 (3.1)	4.3 (3.2)	0.206 (−0.94, 1.3)	0.7
Coherence	7.3 (2.3)	7.2 (2.4)	7.9 (2.1)	7.8 (2.2)	0.008 (−0.58,0.60)	1.0
Emotional rep.	4.0 (2.9)	4.2 (2.9)	4.0 (3.2)	3.2 (2.5)	1.03 (0.13, 1.9)	0.026
B-IPQ composite score	30 (12)	29 (13)	31 (15)	26 (14)	4.2 (−0.57, 8.9)	0.085
HRV coherence	2.8 (1.3)	2.7 (1.3)	2.7 (1.02)	2.7 (1.2)	0.071 (−0.39, 0.53)	0.8
HRV achievement	318 (138)	317 (149)	315 (119)	310 (137)	9.3 (−45, 63)	0.8

Note: K10 = Kessler Psychological Distress Scale. B-IPQ = Brief Illness Perception Questionnaire. HRV = Heart Rate Variability. At 6 mo, 62 participants were in the control group and 66 participants were in the treatment group. Observed means at the baseline and 6 months are reported. * Linear mixed effects model adjusted for baseline outcome variable; age; relationship status; Charlson Comorbidity index; time between trial randomization and treatment; treatment modality; and prescribed medication for anxiety, depression, or both. Covariance matrix of within subject measurements was unstructured.

**Table 3:** Mediation analysis results at 6 months for each potential mediator on K10, n = 128.

	Total Effect			Direct Effect			Indirect Effect		Percentage Mediated
Mediator	Effect Size (95% CI)	SE	*p*	Effect Size (95% CI)	SE	*p*	Effect Size (95% CI)	SE	
Self-efficacy	−3.3 (−5.9, −0.59)	1.3	0.017	−1.6 (−3.9, 0.77)	1.2	0.19	−1.7 (−3.7, −0.17)	0.92	52%
B-IPQ personal control	−3.3 (−6.0, −0.63)	1.3	0.016	−2.7 (−5.4, −0.05)	1.3	0.046	−0.58 (−1.4, −0.001)	0.36	18%
B-IPQ emotional representation	−3.4 (−6.1, −0.79)	1.3	0.011	−1.9 (−4.3, 0.38)	1.2	0.099	−1.5 (−3.5, −0.090)	0.87	44%

*Note: B-IPQ = Brief Illness Perception Questionnaire. K10 = Kessler Non-Specific Psychological Distress Scale. SE = standard error; CI = confidence interval—all CIs were obtained using bootstrapping (n = 10,000). Total, direct, and indirect effects obtained using the PROCESS macro for SPSS. The effect size is the adjusted treatment difference (i.e., non-standardized treatment difference).*

**Table 4:** Moderated mediation analyses for treatment modality, comparing radical prostatectomy and radiotherapy.

Mediator	Effect size	SE	Lower CI	Upper CI
Self-efficacy	−2.3	1.6	−0.52	5.8
B-IPQ personal control	−0.64	0.59	−1.9	0.37
B-IPQ emotional representation	1.6	1.4	−1.3	4.4

*Note: SE = standard error; CI = confidence interval.*

## Data Availability

Data from this study are available to researchers through a data access process in compliance with patient privacy and protection research acts (NSHA Research Ethics Board).

## References

[1] Sung H., Ferlay J., Siegel R.L., Laversanne M., Soerjomataram I., Jemal A., Bray F. (2021). Global Cancer Statistics 2020: GLOBOCAN Estimates of Incidence and Mortality Worldwide for 36 Cancers in 185 Countries. CA A Cancer J. Clin..

[2] Canadian Cancer Statistics Advisory in collaboration with the Canadian Cancer Society, Statistics Canada and the Public Health Agency of Canada (2022). Canadian Cancer Statistics: A 2022 Special Report on Cancer Prevalence.

[3] James N.D., Tannock I., N’Dow J., Feng F., Gillessen S., Ali S.A., Trujillo B., Al-Lazikani B., Attard G., Bray F. (2024). The Lancet Commission on prostate cancer: Planning for the surge in cases. Lancet.

[4] Canadian Cancer Statistics Advisory Committee in collaboration with the Canadian Cancer Society, Statistics Canada and the Public Health Agency of Canada (2021). Canadian Cancer Statistics.

[5] Crump C., Stattin P., Brooks J.D., Sundquist J., Bill-Axelson A., Edwards A.C., Sundquist K., Sieh W. (2023). Long-term Risks of Depression and Suicide Among Men with Prostate Cancer: A National Cohort Study. Eur. Urol..

[6] Fervaha G., Izard J.P., Tripp D.A., Rajan S., Leong D.P., Siemens D.R. (2019). Depression and prostate cancer: A focused review for the clinician. Urol. Oncol. Semin. Orig. Investig..

[7] Ilie G., Rutledge R., Sweeney E. (2019). Anxiety and depression symptoms in adult males in Atlantic Canada with or without a lifetime history of prostate cancer. Psycho-Oncology.

[8] Moodie L., Ilie G., Rutledge R., Andreou P., Kirkland S. (2020). Assessment of Current Mental Health Status in a Population-Based Sample of Canadian Men With and Without a History of Prostate Cancer Diagnosis: An Analysis of the Canadian Longitudinal Study on Aging (CLSA). Front. Psychiatry.

[9] Menichetti J., Villa S., Magnani T., Avuzzi B., Bosetti D., Marenghi C., Morlino S., Rancati T., Van Poppel H., Salvioni R. (2016). Lifestyle interventions to improve the quality of life of men with prostate cancer: A systematic review of randomized controlled trials. Crit. Rev. Oncol./Hematol..

[10] Dovey Z., Horowitz A., Waingankar N. (2023). The influence of lifestyle changes (diet, exercise and stress reduction) on prostate cancer tumour biology and patient outcomes: A systematic review. BJUI Compass.

[11] Martín-Núñez J., Heredia-Ciuró A., Valenza-Peña G., Granados-Santiago M., Hernández-Hernández S., Ortiz-Rubio A., Valenza M.C. (2023). Systematic review of self-management programs for prostate cancer patients, a quality of life and self-efficacy meta-analysis. Patient Educ. Couns..

[12] Ilie G., Rendon R., Mason R., MacDonald C., Kucharczyk M.J., Patil N., Bowes D., Bailly G., Bell D., Lawen J. (2023). A Comprehensive 6-mo Prostate Cancer Patient Empowerment Program Decreases Psychological Distress Among Men Undergoing Curative Prostate Cancer Treatment: A Randomized Clinical Trial. Eur. Urol..

[13] Ilie G., MacDonald C., Richman H., Rendon R., Mason R., Nuyens A., Bailly G., Bell D., Patil N., Bowes D. (2023). Assessing the Efficacy of a 28-Day Comprehensive Online Prostate Cancer Patient Empowerment Program (PC-PEP) in Facilitating Engagement of Prostate Cancer Patients in Their Survivorship Care: A Qualitative Study. Curr. Oncol..

[14] Audulv Å., Packer T., Hutchinson S., Roger K.S., Kephart G. (2016). Coping, adapting or self-managing—What is the difference? A concept review based on the neurological literature. J. Adv. Nurs..

[15] Foster C., Brown J., Killen M., Brearley S. (2007). The NCRI Cancer Experiences Collaborative: Defining self management. Eur. J. Oncol. Nurs..

[16] Dwarswaard J., Bakker E.J.M., van Staa A., Boeije H.R. (2015). Self-management support from the perspective of patients with a chronic condition: A thematic synthesis of qualitative studies. Health Expect..

[17] Bandura A. (1986). Social Foundations of Thought and Action: A Social Cognitive Theory.

[18] Leventhal H., Rachman S. (1980). The common sense representation of illness danger. Contributions to Medical Psychology.

[19] Bandura A. (1997). Self-Efficacy: The Exercise of Control.

[20] Chirico A., Lucidi F., Merluzzi T., Alivernini F., Laurentiis M., Botti G., Giordano A. (2017). A meta-analytic review of the relationship of cancer coping self-efficacy with distress and quality of life. Oncotarget.

[21] Merluzzi T.V., Nairn R.C., Hegde K., Martinez Sanchez M.A., Dunn L. (2001). Self-efficacy for coping with cancer: Revision of the Cancer Behavior Inventory (version 2.0). Psycho-Oncology.

[22] Bandura A., Snyder C.R., Lopez S.J. (2008). An agentic perspective on positive psychology. Positive Psychology: Exploring the Best in People.

[23] DiClemente R., Nowara A., Shelton R., Wingood G. (2019). Need for Innovation in Public Health Research. Am. J. Public. Health.

[24] Farley H. (2019). Promoting self-efficacy in patients with chronic disease beyond traditional education: A literature review. Nurs. Open.

[25] Broadbent E., Petrie K.J., Main J., Weinman J. (2006). The Brief Illness Perception Questionnaire. J. Psychosom. Res..

[26] Leventhal H., Phillips L.A., Burns E. (2016). The Common-Sense Model of Self-Regulation (CSM): A dynamic framework for understanding illness self-management. J. Behav. Med..

[27] Zhang Z., Yang L., Xie D., Wang Y., Bi L., Zhang T., Wang D., Shi H., Li G., Yu D. (2019). Illness perceptions are a potential predictor of psychological distress in patients with non-muscle-invasive bladder cancer: A 12-month prospective, longitudinal, observational study. Psychol. Health Med..

[28] Tian X., Tang L., Yi L.J., Qin X.P., Chen G.H., Jiménez-Herrera M.F. (2022). Mindfulness Affects the Level of Psychological Distress in Patients with Lung Cancer via Illness Perception and Perceived Stress: A Cross-Sectional Survey Study. Front. Psychol..

[29] Akin-Odanye E.O., Ogo C.N., Sulaiman F.A., Suleiman L., Ogunsanya M.E., Odedina F.T. (2021). Examining the influence of illness perception and financial toxicity on the quality of life of prostate cancer patients. Afr. J. Urol..

[30] Ashley L., Marti J., Jones H., Velikova G., Wright P. (2015). Illness perceptions within 6 months of cancer diagnosis are an independent prospective predictor of health-related quality of life 15 months post-diagnosis. Psycho-Oncology.

[31] Breland J.Y., Wong J.J., McAndrew L.M. (2020). Are Common Sense Model constructs and self-efficacy simultaneously correlated with self-management behaviors and health outcomes: A systematic review. Health Psychol. Open.

[32] McCraty R., Atkinson M., Tiller W.A., Rein G., Watkins A.D. (1995). The effects of emotions on short-term power spectrum analysis of heart rate variability. Am. J. Cardiol..

[33] McCraty R., Zayas M.A. (2014). Cardiac coherence, self-regulation, autonomic stability, and psychosocial well-being. Front. Psychol..

[34] McCraty R., Shaffer F. (2015). Heart Rate Variability: New Perspectives on Physiological Mechanisms, Assessment of Self-regulatory Capacity, and Health Risk. Glob. Adv. Health Med..

[35] Burge L., Ilie G., MacDonald C., Riel H., Rutledge R.D.H. (2023). Changes in Stress Reduction Following a 28-Day Prostate Cancer Patient Empowerment Program (PC-PEP) among Prostate Cancer Survivors. Curr. Oncol..

[36] Burch J.B., Ginsberg J.P., McLain A.C., Franco R., Stokes S., Susko K., Hendry W., Crowley E., Christ A., Hanna J. (2020). Symptom Management Among Cancer Survivors: Randomized Pilot Intervention Trial of Heart Rate Variability Biofeedback. Appl. Psychophysiol. Biofeedback.

[37] Preacher K.J., Rucker D.D., Hayes A.F. (2007). Addressing Moderated Mediation Hypotheses: Theory, Methods, and Prescriptions. Multivar. Behav. Res..

[38] Donovan J.L., Hamdy F.C., Lane J.A., Mason M., Metcalfe C., Walsh E., Blazeby J.M., Peters T.J., Holding P., Bonnington S. (2016). Patient-Reported Outcomes after Monitoring, Surgery, or Radiotherapy for Prostate Cancer. N. Engl. J. Med..

[39] Harris P.A., Taylor R., Minor B.L., Elliott V., Fernandez M., O’Neal L., McLeod L., Delacqua G., Delacqua F., Kirby J. (2019). The REDCap consortium: Building an international community of software platform partners. J. Biomed. Inform..

[40] Kessler R.C., Barker P.R., Colpe L.J., Epstein J.F., Gfroerer J.C., Hiripi E., Howes M.J., Normand S.L., Manderscheid R.W., Walters E.E. (2003). Screening for Serious Mental Illness in the General Population. Arch. Gen. Psychiatry.

[41] Andrews G., Slade T. (2001). Interpreting scores on the Kessler Psychological Distress Scale (K10). Aust. N. Z. J. Public Health.

[42] Thakre M., Sathe H., Talapalliwar M. (2023). Psychometric properties of Kessler’s Psychological Distress Scale (K10) in cancer patients. Arch. Ment. Health.

[43] Lorig K.R., Sobel D.S., Ritter P.L., Laurent D., Hobbs M. (2001). Effect of a self-management program on patients with chronic disease. Eff. Clin. Pract..

[44] Ritter P.L., Lorig K. (2014). The English and Spanish Self-Efficacy to Manage Chronic Disease Scale measures were validated using multiple studies. J. Clin. Epidemiol..

[45] Broadbent E., Wilkes C., Koschwanez H., Weinman J., Norton S., Petrie K.J. (2015). A systematic review and meta-analysis of the Brief Illness Perception Questionnaire. Psychol. Health.

[46] Kuiper H., van Leeuwen C.M.C., Stolwijk-Swüste J.M., Post M.W.M. (2021). Reliability and validity of the Brief Illness Perception Questionnaire (B-IPQ) in individuals with a recently acquired spinal cord injury. Clin. Rehabil..

[47] Zhang N., Fielding R., Soong I., Chan K.K., Lee C., Ng A., Sze W.K., Tsang J., Lee V., Lam W.W. (2017). Psychometric assessment of the Chinese version of the brief illness perception questionnaire in breast cancer survivors. PLoS ONE.

[48] Tiller W.A., McCraty R., Atkinson M. (1996). Cardiac coherence: A new, noninvasive measure of autonomic nervous system order. Altern. Ther. Health Med..

[49] Kurian C.J., Leader A.E., Thong M.S.Y., Keith S.W., Zeigler-Johnson C.M. (2018). Examining relationships between age at diagnosis and health-related quality of life outcomes in prostate cancer survivors. BMC Public Health.

[50] Rice S.M., Oliffe J.L., Kelly M.T., Cormie P., Chambers S., Ogrodniczuk J.S., Kealy D. (2018). Depression and Prostate Cancer: Examining Comorbidity and Male-Specific Symptoms. Am. J. Mens. Health.

[51] Luckenbaugh A.N., Wallis C.J.D., Huang L.C., Wittmann D., Klaassen Z., Zhao Z., Koyama T., Laviana A.A., Conwill R., Goodman M. (2022). Association between Treatment for Localized Prostate Cancer and Mental Health Outcomes. J. Urol..

[52] Spiker R.L., Cockerham W.C., Dingwall R., Quah S.R. (2014). Mental Health and Marital Status. The Wiley Blackwell Encyclopedia of Health, Illness, Behavior, and Society.

[53] IBM (2021). IBM SPSS Statistics for Macintosh, Version 28.0.

[54] Hayes A.F. (2017). Introduction to Mediation, Moderation, and Conditional Process Analysis: A Regression-Based Approach.

[55] Rijnhart J.J.M., Twisk J.W.R., Chinapaw M.J.M., de Boer M.R., Heymans M.W. (2017). Comparison of methods for the analysis of relatively simple mediation models. Contemp. Clin. Trials Commun..

[56] Vo T.T., Superchi C., Boutron I., Vansteelandt S. (2020). The conduct and reporting of mediation analysis in recently published randomized controlled trials: Results from a methodological systematic review. J. Clin. Epidemiol..

[57] Calvo-Schimmel A., Qanungo S., Newman S., Sterba K. (2021). Supportive care interventions and quality of life in advanced disease prostate cancer survivors: An integrative review of the literature. Can. Oncol. Nurs. J..

[58] Martín-Núñez J., Raya-Benítez J., López-López L., Calvache-Mateo A., Heredia-Ciuró A., Navas-Otero A., Valenza M.C. (2023). Efficacy in urinary symptom burden, psychological distress, and self-efficacy of education-enhanced interventions in prostate cancer patients: A systematic review and meta-analyses. Support Care Cancer.

[59] Yang R., Lu Z., Gu X., Dai B. (2021). The Effect of an Information Support Program on Self-Efficacy of Prostate Cancer Patients during Hormonal Therapy. Asia-Pac. J. Oncol. Nurs..

[60] Muzii B., Di Bello F., Carraturo F., Di Perna T., Califano G., Morra S., Mangiapia F., Scandurra C., Giuliani L., Celentano G. (2023). Mental Health of Prostate Cancer Patients: Content Review on YouTubeTM. Int. J. Environ. Res. Public Health.

[61] Dziak J.J., Dierker L.C., Abar B. (2018). The interpretation of statistical power after the data have been gathered. Curr. Psychol..

[62] Baron R.M., Kenny D.A. (1986). The moderator–mediator variable distinction in social psychological research: Conceptual, strategic, and statistical considerations. J. Pers. Soc. Psychol..

[63] Hayes A.F., Montoya A.K., Rockwood N.J. (2017). The Analysis of Mechanisms and Their Contingencies: PROCESS versus Structural Equation Modeling. Australas. Mark. J..

[64] Silver J.K., Baima J. (2013). Cancer Prehabilitation. Am. J. Phys. Med. Rehabil..

